# Leydig cell metabolic disorder act as a new mechanism affecting for focal spermatogenesis in Klinefelter syndrome patients: a real world cross-sectional study base on the age

**DOI:** 10.3389/fendo.2023.1266730

**Published:** 2023-11-01

**Authors:** Huang Liu, Zhenhui Zhang, Yong Gao, Hai Lin, Zhiyong Zhu, Houbin Zheng, Wenjing Ye, Zefang Luo, Zhaohui Qing, Xiaolan Xiao, Lei Hu, Yu Zhou, Xinzong Zhang

**Affiliations:** ^1^ Department of Andrology, National Health Commission (NHC) Key Laboratory of Male Reproduction and Genetics, Guangdong Provincial Reproductive Science Institute (Guangdong Provincial Fertility Hospital), Human Sperm Bank of Guangdong Province, Guangzhou, China; ^2^ Reproductive Medicine Center, Shunde Hospital, Southern Medical University (The First People’s Hospital of Shunde), Foshan, China; ^3^ Department of Reproductive Medicine Center, Guangdong Provincial Key Laboratory of Reproductive Medicine, Guangdong Provincial Clinical Research Center for Objective and Gynecological Diseases, Sun Yat-sen University First Affiliated Hospital, Guangzhou, China; ^4^ Reproductive Medicine Center, National Health Commission (NHC) Key Laboratory of Male Reproduction and Genetics, Guangdong Provincial Reproductive Science Institute (Guangdong Provincial Fertility Hospital), Human Sperm Bank of Guangdong Province, Guangzhou, China; ^5^ Department of Anesthesiology, National Health Commission (NHC) Key Laboratory of Male Reproduction and Genetics, Guangdong Provincial Reproductive Science Institute (Guangdong Provincial Fertility Hospital), Human Sperm Bank of Guangdong Province, Guangzhou, China

**Keywords:** Klinefelter syndrome, Leydig cell, metabolic disorder, age, microscopic testicular sperm extraction, spermatogenic

## Abstract

**Background:**

Klinefelter’s syndrome (KS) was once considered infertile due to congenital chromosomal abnormalities, but the presence of focal spermatozoa changed this. The key to predict and promote spermatogenesis is to find targets that regulate focal spermatogenesis.

**Objective:**

To explore the trend of fertility changes in KS patients at different ages and identify potential therapeutic targets.

**Methods:**

Bibliometric analysis was used to collect clinical research data on KS from the Web of Science Core Collection (WoSCC) from 1992 to 2022. A cross-sectional study was conducted on 75 KS patients who underwent microscopic testicular sperm extraction (mTESE) from 2017 to 2022 in the real world. The reproductive hormones, testicular histopathology, androgen receptors, insulin-like factor 3 (INSL3) receptors and sperm recovery rate (SRR) were analyzed.

**Results:**

Male infertility, dysplasia, Sertoli cells, Leydig cells, testosterone and spermatogenesis were the research focuses related to KS. Luteinizing hormone (LH), testosterone, and INSL3 were evaluation indicators of Leydig cell function that fluctuate with age. Testosterone and LH peaked at ages 13-19 and 30-45, while INSL3 only peaked at ages 13-19. 27 patients (27/75) recovered sperm through mTESE and experienced SRR peaks at the ages of 20, 28, 34, and 37. The SRR of fibrosis patients was 46.15%, fatty degeneration was 7.14%, and melanosis was 40.00%. The INSL3 and androgen receptors were highly expressed and roughly balanced in focal spermatogenesis.

**Conclusion:**

Abnormal metabolism of Leydig cells led to imbalanced expression of INSL3 and androgen receptors, which might be a potential target for spermatogenesis in KS.

## 1 Introduction

Klinefelter syndrome (KS), the most common sex chromosomal abnormality, was first described by Harry F. Klinefelter (1) in 1942 as a clinical entity presenting with features of primary hypogonadism, and the disorder, confirmed by Jacobs (2) through cytogenetics, was demonstrated to be caused by one or more X chromosomes inherited from the paternal or maternal chromosome. The classical clinical manifestations of KS are small firm testes, eunuchoidism stature, sexual dysfunction, infertility and gynecomastia, sometimes accompanied by hyperglycemia, obesity, osteoporosis, cognitive dysfunction and psychological problems, but the phenotype can vary from the typical one with androgen deficiency to a normally virilized group (3, 4). The prevalence of the syndrome, ranging between 0.1% and 0.2% in newborn male infants, increases to approximately 3% in infertile males and to 10–12% among azoospermic patients, and the incidence tends to increase yearly according to a recent study (4, 5).

Nevertheless, due to the variability of the phenotype or to the lack of typical symptoms in prepuberty, approximately two-thirds of Klinefelter patients are not confirmed during their whole life, and the rest can sometimes be diagnosed mainly because of infertility in the reproductive domain (3). Assisted reproductive technology, especially intracytoplasmic sperm injection (ICSI) combined with microdissection testicular sperm extraction (mTESE), is the core therapy for KS patients who suffer from azoospermia to seize the hope of having their own offspring (6). Unfortunately, there are still many problems in the existing diagnosis and treatment of KS. On the one hand, the failure of generative cell function starts from puberty, but the low diagnostic rate may deprive us of the best timeline for testicular sperm extraction (TESE) and fertility preservation action (3, 7, 8). On the other hand, although the fluctuation of serum hormones such as testosterone, follicle-stimulating hormone and luteinizing hormone is helpful in evaluating spermatogenesis, there are discrepant data and opinions on whether these indexes are capable of accurately predicting the success of mTESE in KS (9, 10). From the aspect of histopathology, KS is characterized by progressive hyalinization of the seminiferous tubules of the testis and loss of spermatogonia, accompanied by the hyperplasia of Leydig cells, which is contradictory to the absence of androgen levels in adult patients with KS (11, 12). The majority of previous studies of the various cell types that exist in the testis have focused on the degeneration of germ cells and Sertoli cells in the field of KS (7, 13–15). Although the disturbed maturation of Leydig cells has attracted further attention in recent research on KS, there is still little conclusive evidence of the pathophysiological mechanism and treatment methods based on Leydig cells (16, 17). An in-depth understanding of the mechanisms of testicular cell failure and its timeline is supposed to help better inform the constant debate regarding the related factors affecting therapeutic outcome in KS and to help further understand the clinical heterogeneity in different age groups of KS.

Taken together, a comprehensive assessment of fertility in KS patients is essential for patient counseling, treatment decision-making and therapeutic benefits. In this study, we preliminarily summarized the emerging trends and the cluster of studies on Leydig cells in patients with KS using a bibliometric analysis of the scientific landscape, which is a qualitative and quantitative tool frequently applied to systematically study the characteristics of global scientific literature and to build the knowledge frame in specific fields (18, 19). The interesting results of the visual landscape include numerous randomized controlled trials (RCTs), in which recruited participants meet the specific inclusion criteria and are monitored with predefined protocols.

Therefore, a real-world study was then conducted to consistently demonstrate whether the results could be extended to an actual clinical environment. In real-world clinical settings, global multidisciplinary clinicians have been actively exploring multiple factors and seeking means to predict the testicular function of patients with KS at different ages. At present, there is a lack of specific predictive methods for the occurrence of focal spermatogenesis in the testicles of KS patients. However, in clinical practice, it has been found that the probability of focal spermatogenesis varies among KS patients at different ages. Leydig cells constitute the microenvironment of spermatogenesis, and we hypothesized that metabolic function may have potential value in these individuals’ focal spermatogenesis, and the sign of Leydig cell metabolism disorder or advanced failure in the testis among different age groups could help better understand the dynamic progression of KS.

## 2 Method

### 2.1 Literature retrieval

**Figure 1:**
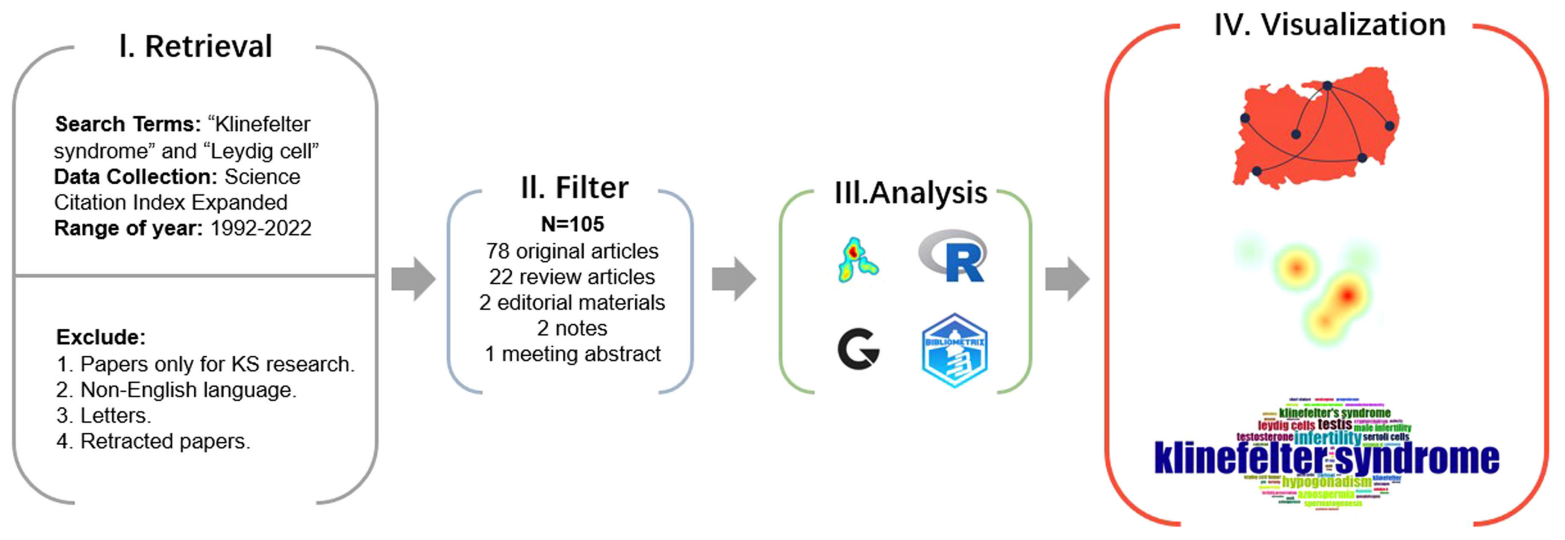
Process of bibliometric and visual analysis.

Relevant bibliometric data on KS and Leydig cells were collected and identified using the Web of Science Core Collection (WoSCC) database for the preliminary review (**Figure 1**). The WoSCC is a famous scientific database with a high reputation in the global academic community and is regarded as one of the most available sources of the bibliometric analysis of scientific publications worldwide, breaking the barriers of interaction between disciplines, journals and publishers (20–22). To capture current research trends of morphological and functional changes of Leydig cells in KS, we limited the search of relevant documents to the “article title, abstract and keywords” of publications in the WoSCC on the basis of the previous literature retrieval strategy, applying the main search terms of “Klinefelter syndrome” and “Leydig cell” with the operation method “AND” on 17 December 2022. Original articles, editorial materials, meeting abstracts (those including relevant information of patients), notes and review articles in Science Citation Index Expanded (SCIE) were incorporated into our study. To limit the potential impact of irrelevant or duplicate titles, the researchers conducted duplicate detection, and the documents were filtered for relevance and uniqueness. Additionally, only papers in English with raw data of titles, authors, abstracts, keywords, and references from WoSCC were downloaded and saved as plain text files for further data analysis.

### 2.2 Visual analysis

The preprocessed data were then analyzed using VOSviewer version 1.6.18, Scimago Graphica version 1.0.26 (https://www.graphica.app), R software version 4.2.1 (https://www.r-project.org) with the bibliometrix R package (https://www.bibliometrix.org) and Microsoft Excel Version 2019.

R software and the core R package were applied to fetch the information from the raw files. VOSviewer and SCImage Graphics were then used to plot visual bibliometric maps to present the collaboration relationship between countries, institutions and authors of highly cited literature, the academic influence of authors, and the citation and reference situation. In addition, VOSviewer was also applied to sort out the keywords with high co-occurrence frequency into several clusters and to reveal the research focuses and trends.

### 2.3 Reproductive hormone characteristics in literature data

In addition, additional sub-analyses were carried out for the ten experimental articles including complete clinical information of patients on the indexes with potential predictive significance in the shape, quantity or function of Leydig cells to investigate the influence of multiple factors on the Leydig cells of patients with KS. Then, according to the literature data, the mean values of testosterone, luteinizing hormone and INSL3 of the subjects sorted at different ages, and the sample size were summarized and statistically analyzed.

### 2.4 Real-world research

#### 2.4.1 Patient data

**Figure 2:**
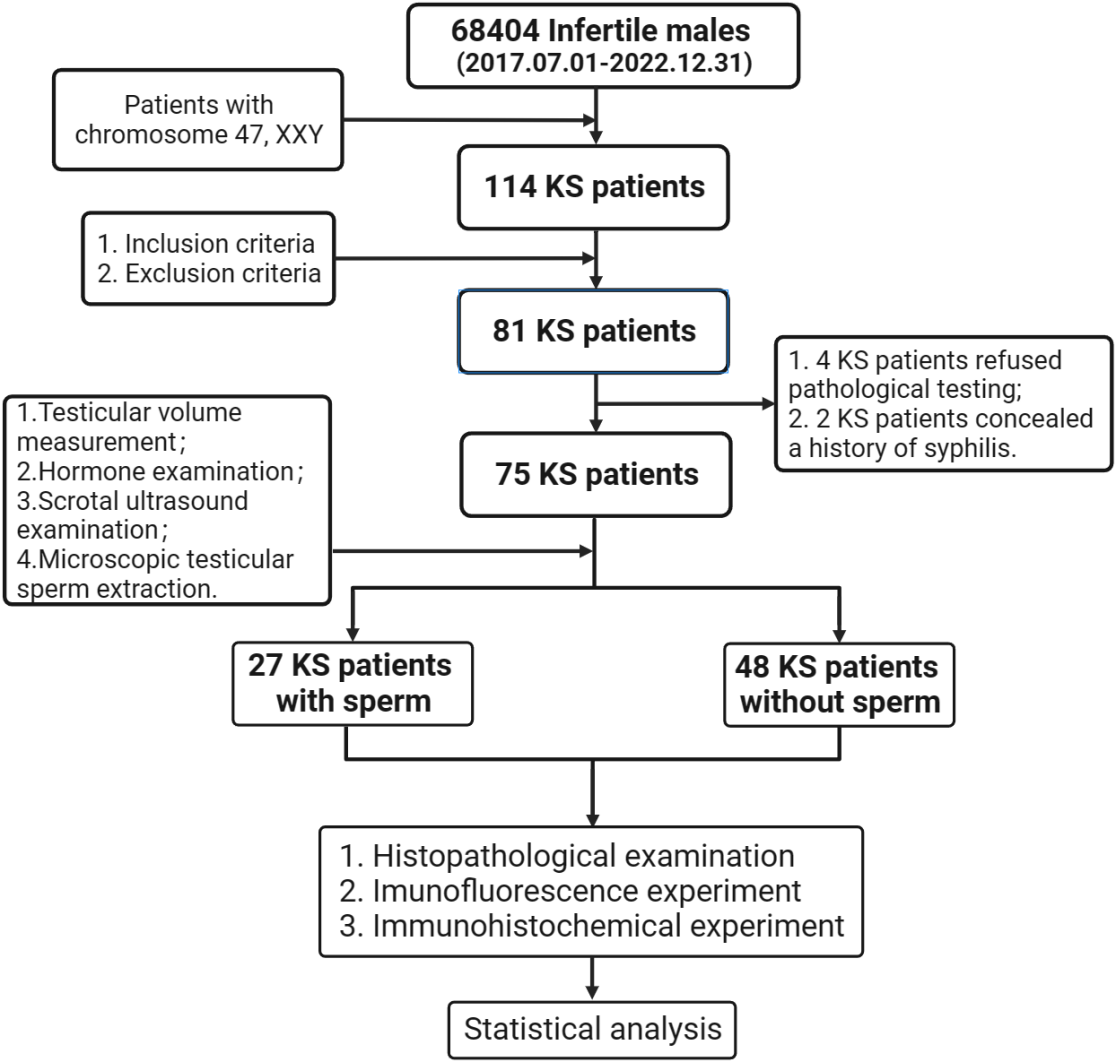
Overview of the real world research.

After strictly screening from the 68404 infertile males attended at Guangdong Provincial Reproductive Hospital (Guangdong Provincial Institute of Reproductive Sciences) from July 2017 to December 2022, we eventually enrolled 75 KS patients who were hospitalized for testicular incision and microscopic sperm extraction in this study (**Figure 2**). All patients were operated on after signing the informed consent form. The age of onset, course of disease, drugs used during treatment, and sexual hormone level before and after treatment were recorded in detail, physical examination was performed, and scrotal color ultrasound was reviewed before the operation. This study was approved by the Ethics Committee of Guangdong Provincial Reproductive Science Institute (Guangdong Provincial Fertility Hospital) (Approval No.2021(08)) and had been registered through the Chinese Clinical Trial Registry (https://www.chictr.org.cn/) with the registration number (ChiCTR2200060463).

#### 2.4.2 Inclusion criteria

① According to the karyotype diagnosis of 47, XXY; ② Be able to cooperate with inspection; ③ Willing to accept and able to tolerate mTESE operation; ④ Complete withdrawal from drug treatment for 3 months or more before operation; ⑤ Able to complete sexual intercourse and obtain semen.

#### 2.4.3 Exclusion criteria

① AZF gene deletion; ② Combined with genital tract infection; ③ Combined with systemic diseases; ④ Combined with varicocele; ⑤ Those who still have drug intervention within three months before operation; ⑥ Combined tumor, especially tumor of reproductive organs; ⑦ He has received radiotherapy and chemotherapy. ⑧ Stay up late for a long time or have bad habits such as smoking, alcohol and drug use; ⑨ There are familial hereditary diseases; ⑩ Sexual and ejaculatory dysfunction.

#### 2.4.4 Method

##### 2.4.4.1 Testicular volume

The testicular volume measurement quantum model (size: 1, 2, 3, 4, 5, 6, 8, 10, 12, 15, 20, >25 mL) was used to measure the testicular volume, and the testicular volume was matched with the color Doppler ultrasound data (23).

##### 2.4.4.2 Hormone

An automatic chemiluminescence instrument (Roche reagent kit) was used to measure the concentration of sex hormones in peripheral blood, including follicle-stimulating hormone (Roche reagent kit 11775863122), luteinizing hormone (Roche reagent kit 11732234122), testosterone (Roche reagent kit 05200067190) and estradiol (Roche reagent kit 06656021190).

##### 2.4.4.3 Microscopic testicular sperm extraction

All patients were under general anesthesia for tracheal intubation, and a ZEISS S88 ultrahigh-definition operating microscope was used for testicular incision and microscopic sperm extraction. During the operation, the equatorial plane of the testis was taken to cut the white membrane of the testis, expose the tissue in the testis, carefully distinguish the seminiferous tubules, interstitial tissue and capillaries, etc., and the relatively formed seminiferous tubules were extracted and sent to a Nikon inverted microscope to tear up and check whether there was sperm. If no sperm are found in one testicle, cut the other testicle to look for sperm and thoroughly determine whether there are sperm. At the same time, a small amount of testicular tissue was taken for pathological examination during the operation. Photographs and videos were used to record the condition and operation process of testicular tissue during the operation.

##### 2.4.4.4 Scrotal ultrasound examination

All patients were placed in the supine position at room temperature (26 °C), and a 7-14 Hz probe was used to scan both sides of the scrotum longitudinally and transversely. The length, width and thickness of the testicle were recorded, and the following formula was applied: testicular volume=length × wide diameter × thickness × 0.71 (24).

##### 2.4.4.5 Histopathological examination

The testicular tissue removed under sterile conditions during the operation was placed in Bonn’s immersion and sent for histopathological examination on the same day. The condition of spermatogenic cells in the tissue was scored by the Johnson scoring method (25).

##### 2.4.4.6 Immunofluorescence experiment

Paraffin sections of testicular tissue obtained from the mTESE operation were dewaxed and dehydrated and then placed in a microwave oven for antigen repair. Primary antibodies (Anti -INSL3 Rabbit pAb, GB113558-100, Servicebio, 1: 3000) and secondary antibodies (HRP labeled goat anti rabbit secondary antibody, GB23303, Servicebio, 1:200) were added for incubation, and DAPI was added dropwise for staining. Follow the steps below to conduct the experiment: ① Paraffin section dewaxing to water. ② Antigen repair. ③ Blocking endogenous peroxidase. ④ Serum blocking. ⑤ Adding Primary antibodies(Anti -INSL3 Rabbit pAb, GB113558-100, Servicebio, 1: 3000). ⑥ Adding secondary antibody (HRP labeled goat anti rabbit secondary antibody, GB23303, Servicebio, 1:200). ⑦ DAB color rendering. ⑧ Recombinant staining of cell nucleus. ⑨ Dehydration and sealing. ⑩ Microscopic examination. A self-luminescent quenching agent was added, and then the seal was observed by fluorescence microscopy, and images were collected. The ultraviolet excitation wavelength of DAPI is 330-380 nm, the emission wavelength is 420 nm, and it emits blue light; FITC excitation wavelength 465-495 nm, emission wavelength 515-555 nm, green light; CY3 excitation wavelength 510-560, emission wavelength 590 nm, emitting red light. The results showed that the nucleus stained by DAPI was blue under ultraviolet excitation, and the positive expression was red or green light labeled by corresponding fluorescein.

##### 2.4.4.7 Immunohistochemical experiment

Paraffin sections of testicular tissue obtained from mTESE surgery were dewaxed and dehydrated, placed in a microwave oven for antigen repair, blocking endogenous peroxidase, added to serum to seal the tissue and incubated with the first and second antibodies. Finally, DAB staining solution was added to control color development so that the positive cells were stained brown. Finally, hematoxylin solution was used to restain the nucleus so that the nucleus was stained blue, and the dehydrated seal was placed under a white light microscope to interpret the results. The stained nucleus was blue, and the positive expression of DAB was brown.

#### 2.4.5 Statistical analysis

The data were statistically analyzed and plotted with OriginLab Origin 2022 (OriginLab Corporation, USA). The counting data were expressed in the form of n or percentage (%). The chi-square test was used for analysis. The mean difference of the measurement data was expressed in the form of plus or minus standard deviation. The K-S test was used to test the homogeneity of variances. When the variances were homogeneous, the t test was used to compare the intergroup or intragroup differences. When the variances were uneven, the U test was used. AVNOA univariate analysis of variance was used to assess the significance of each influencing factor. When *P<0.05*, the difference was statistically significant. The missing follow-up data was not included in our final analysis.

## 3 Results

### 3.1 Bibliometric analysis and visualization

#### 3.1.1 Publications and journals

**Figure 3:**
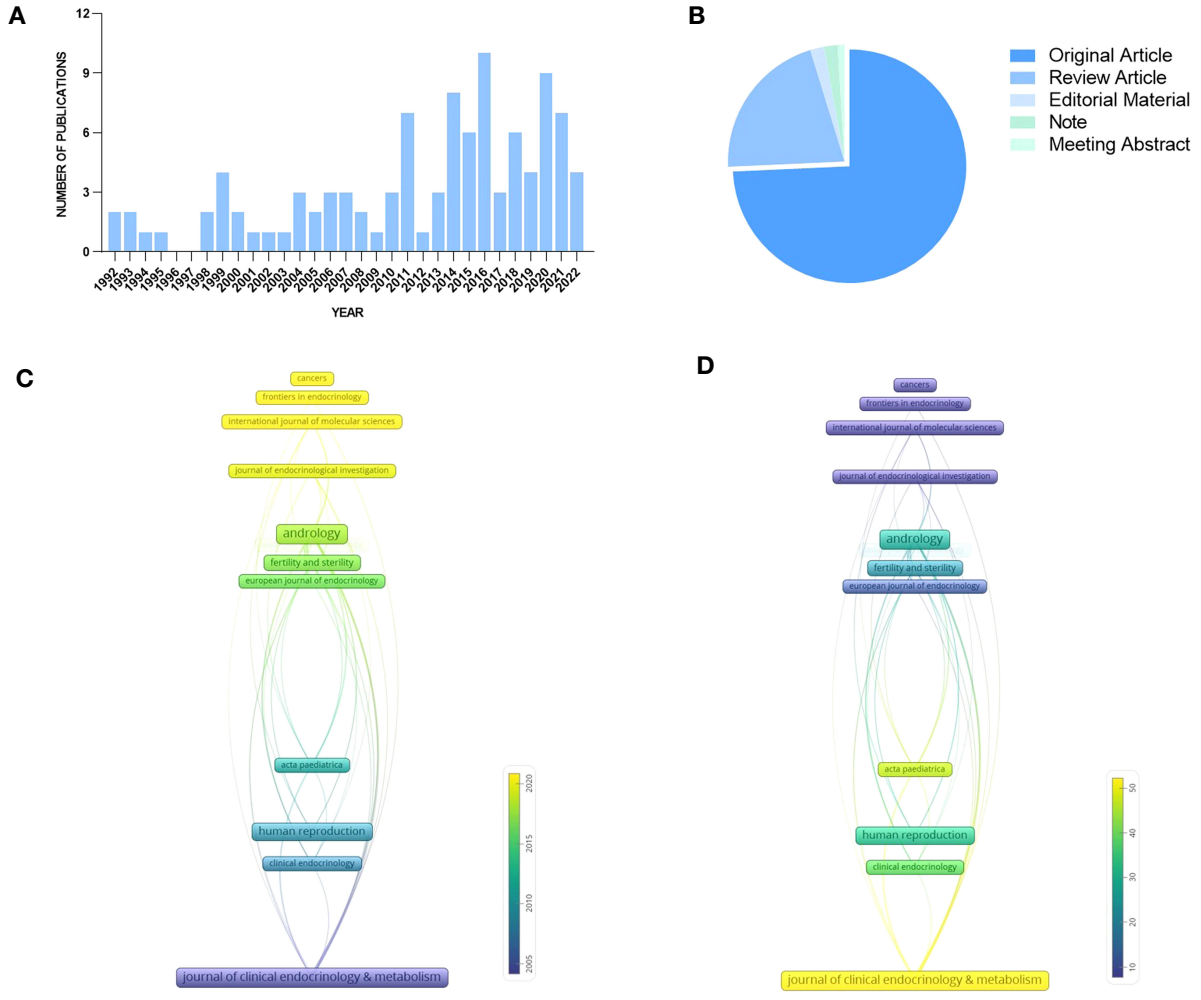
Publications and journals for quantitative analysis: **(A)** Publication output. **(B)** Literature types. **(C)** Average year publication of highly productive journals. **(D)** Average citation scores of highly productive journals.

A total of 105 identified articles met the above retrieval criteria. As shown in **Figure 3A**, the overall quantity of publications is not large, and the annual publication output is unstable from 1992 to 2022, which indicates a lack of research attention to KS and Leydig cells, but the number of documents issued shows a growing trend in the past decade, with two peaks in 2016 and 2020. **Figure 3B** depicts the literature types: 78 original articles (74.286%), 22 review articles (20.952%), 2 editorial materials (1.905%), 2 notes (1.905%), and one meeting abstract (0.952%).

According to our analysis, the published papers about KS and Leydig cells from 1992 to 2022 were mainly disseminated in 66 journals, and the top 15 journals with the largest numbers are summarized in **Table 1**. Journal of Clinical Endocrinology & Metabolism and Andrology were the most productive journals. The impact factors of the top 15 journals in 2022 varied from 3.054 to 7.49, and most of them belong to the United States and the United Kingdom.

**Table 1:** Journals ranked by numbers of articles in the field of Leydig cells in patients with Klinefelter syndrome (Top 15).

	Journal	Articles	Proportion	Region	Import factor(2022)
1	Journal of Clinical Endocrinology & Metabolism	8	7.407	USA	6.134
2	Andrology	8	7.407	USA	4.456
3	Human Reproduction	6	5.556	United Kingdom	6.353
4	Fertility and Sterility	4	3.704	USA	7.49
5	Acta Pediatrica	3	2.778	United Kingdom	4.056
6	Clinical Endocrinology	3	2.778	United Kingdom	3.523
7	Endocrinology	3	2.778	USA	5.051
8	International Journal of Molecular Sciences	3	2.778	USA	6.208
9	Scientific Reports	3	2.778	United Kingdom	4.996
10	American journal of medical genetics. Part C, Seminars in medical genetics	2	1.852	USA	3.359
11	Asian Journal of Andrology	2	1.852	China	3.054
12	European Journal of Endocrinology	2	1.852	United Kingdom	6.558
13	Cancers	2	1.852	Switzerland	6.575
14	Journal of Endocrinological Investigation	2	1.852	Italy	5.467
15	Frontiers in Endocrinology	2	1.852	USA	6.055

The time-based analysis of journal productivity is presented in **Figure 3C**, where the colors denote the average year of all published papers in a journal. The average rate of quoting each paper in the journals is presented in **Figure 3D**, visually indicated by the color evolution from purple to yellow. It can be found that the average publication year of Clinical Endocrinology & Metabolism appears earliest with the highest local citation, which indicated that it had realized the significance of Leydig cells to patients earlier than others. In comparison, the number of published papers from andrology and fertility and sterility appeared to increase between 2015 and 2020, and the two journals also achieved remarkable local citation scores.

#### 3.1.2 Countries and authors

**Figure 4:**
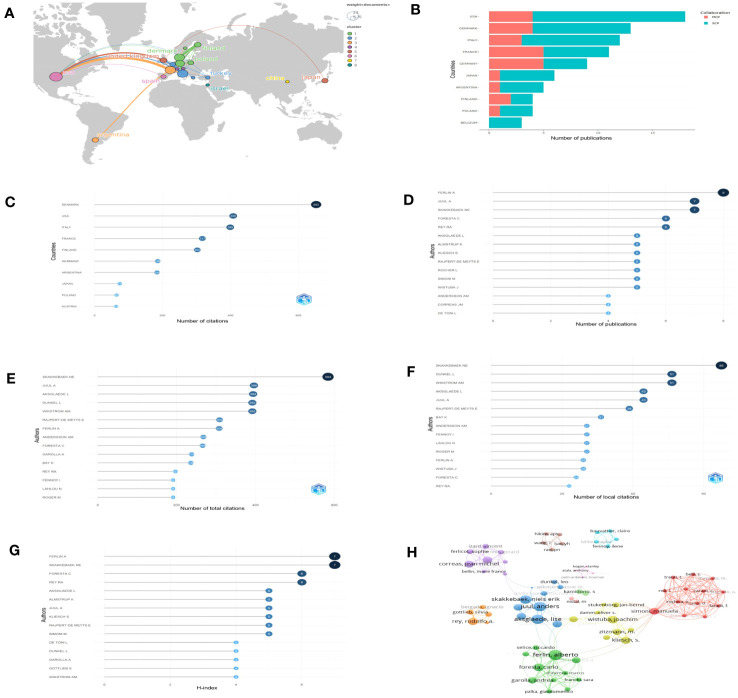
Countries and authors for quantitative analysis: **(A)** The network map of countries. **(B)** Numbers of publications of countries (SCP, single country publication; MCP, multiple country publication. **(C)** Total citations of countries. **(D–G)** The top 15 authors in terms of number of publications, total citations, local citations and H-index. **(H)** Collaboration network.

A total of 23 countries contributed to the published studies on Leydig cells in KS patients. The distribution of published documents and the connection between countries participating in international cooperation are shown on a world map by Scimago Graphica (**Figure 4A**), which indicated that collaborative teams were built between them and that the global research centers were predominantly located in Europe and the United States. According to the findings by R, the United States contributed 18 articles among the top ten countries (**Figure 4B**) active in the relevant studies, and studies from Denmark had the highest number of citations, followed by the United States, Italy, France and Finland. (**Figure 4C**).

Over 508 authors have conducted research on Leydig cells in KS patients. The top 15 authors in terms of number of publications, total citations, local citations and H-index are shown in our results (**Figures 4D–G**) to assess their influence. Among them, Ferlin A was the most prolific author with 8 articles, followed by Juul A (7 articles) and Skakkebaek NE (7 articles). Skakkebaek NE was also cited the most in both total and local citations, 584 times and 65 times, respectively. Juul A was the second most frequently cited author in total citations (396 times), followed by Aksglaede L (394 times), Dunkel L (392 times) and Wikstrom AM (392 times). When applying the h-index to evaluate the influence of authors, the ranking was headed by Ferlin A, which was followed by Skakkebaek NE, Foresta C and Rey RA. The distribution among the authors was also studied and presented by a collaboration network (**Figure 4H**).

#### 3.1.3 Citation and Keywords

**Figure 5:**
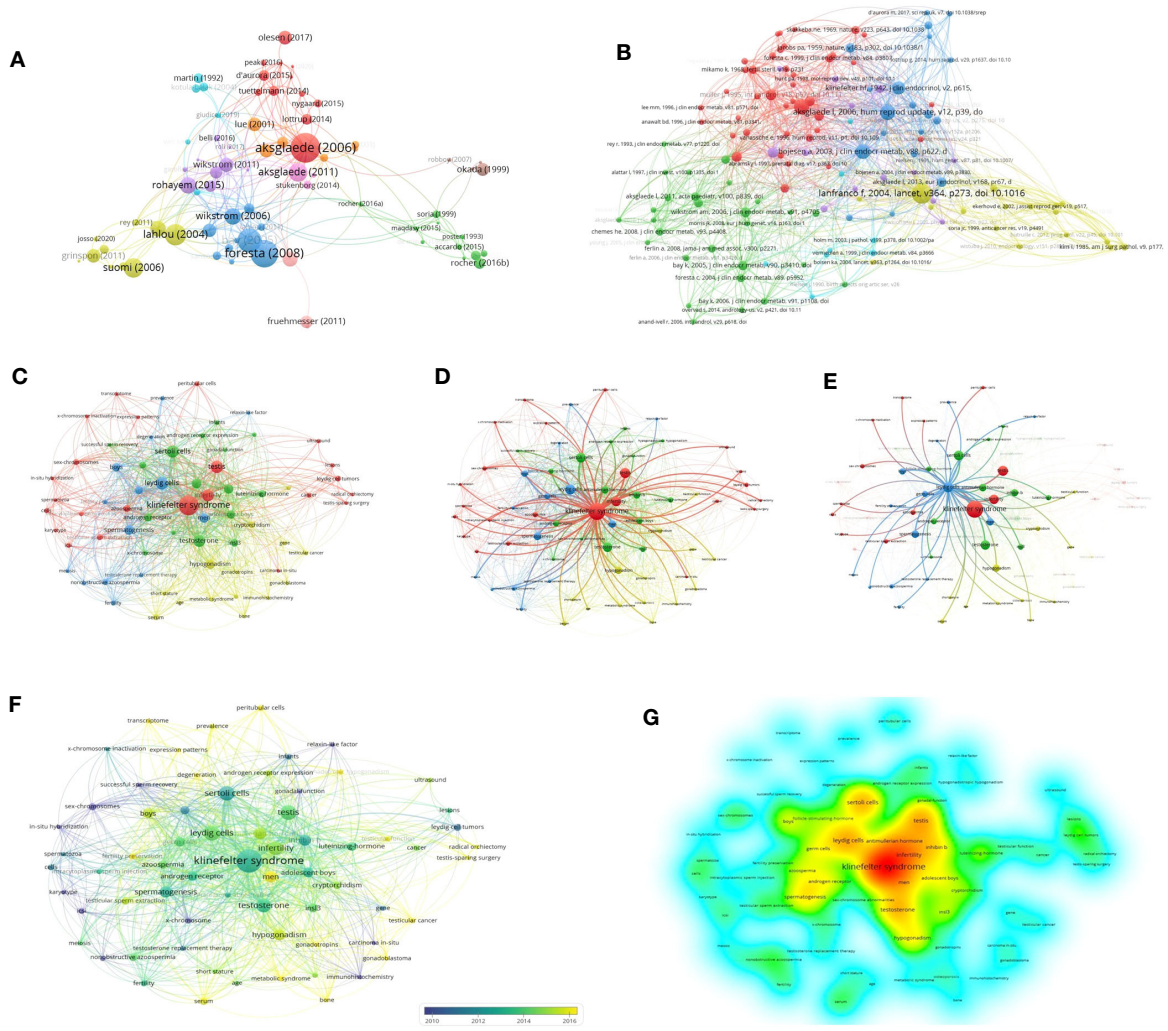
Network and keywords for quantitative analysis: **(A)** Citation Network of documents. **(B)** Cocitation Network of references. **(C)** Network of keywords. **(D)** Network of KS. **(E)** Network of Leydig cells. **(F)** Chronological overlay of keywords. **(G)** Density of keywords.

To analyze the citations of the documents, we present the top 10 documents with the most citations in **Table 2**, which shows that there were 7 articles with more than 10 local citations. Among them, “Natural history of seminiferous tubule degeneration in Klinefelter syndrome” (2006), published by Aksglaede L in Human Reproduction Update, had the most local citations and global citations, up to 25 and 187 times, and it was also the center of the citation network of the documents (**Figure 5A**).

**Table 2:** Top 10 citation documents on Leydig cells in KS patients.

	Document	Year	Title	Local Citations (LC)	Total Citations (TC)	Ratio (TC/LC)	Normalized LC	Normalized TC
1	26, HUM REPROD UPDATE	2006	Natural history of seminiferous tubule degeneration in Klinefelter syndrome.	25	187	13.37	1.92	1.56
2	27, INT J ANDROL	1995	Quantified testicular histology in boys with sex chromosome abnormalities.	15	51	29.41	1.00	1.00
3	28, J CLIN ENDOCR METAB	2004	Inhibin B and anti-Müllerian hormone, but not testosterone levels, are normal in infants with nonmosaic Klinefelter syndrome	14	113	12.39	1.83	1.89
4	29, J CLIN ENDOCR METAB	2006	Serum insulin-like factor 3 levels during puberty in healthy boys and boys with Klinefelter syndrome	13	76	17.11	1.00	0.63
5	30, J CLIN ENDOCR METAB	2005	Insulin-like factor 3 serum levels in 135 normal men and 85 men with testicular disorders: relationship to the luteinizing hormone-testosterone axis	12	133	9.02	1.50	1.41
6	31, CLIN ENDOCRINOL	2007	Establishment of testicular endocrine function impairment during childhood and puberty in boys with Klinefelter syndrome	11	71	15.49	2.75	1.97
7	32, ACTA PAEDIATR	2011	Clinical and biological parameters in 166 boys, adolescents and adults with nonmosaic Klinefelter syndrome: a Copenhagen experience	11	93	11.83	1.88	1.81
8	11, ANDROLOGY-US	2014	Intratesticular testosterone is increased in men with Klinefelter syndrome and may not be released into the bloodstream owing to altered testicular vascularization– a preliminary report	9	36	25.00	3.43	1.68
9	33, BMC GENOMICS	2015	Deregulation of sertoli and leydig cells function in patients with Klinefelter syndrome as evidenced by testis transcriptome analysis	9	41	21.95	2.00	1.33
10	34, J CLIN ENDOCR METAB	2011	Assessment of Leydig and Sertoli cell functions in infants with nonmosaic Klinefelter syndrome: insulin-like peptide 3 levels are normal and positively correlated with LH levels	8	55	14.55	1.37	1.07

Based on a total of 3700 cited references, **Figure 5B** shows the cocitation network between cited references with more than five citations, which reveals that references with the strongest citation bursts were Lanfranco F (2004), Aksglaede L (2006) and Wikstrom AM (2004).

A total of 195 keywords among 3049 keywords were identified from 105 documents after we normalized similar keywords such as “Klinefelter syndrome”, “XXY syndrome” and “Klinefelter’s syndrome” and set the minimum number of occurrences of a keyword to two times to avoid redundancy and synonyms. **Figure 5A** provides an overview of overall trends and interconnections in the field. Klinefelter syndrome, infertility, testis, testosterone, spermatogenesis, Sertoli cells and Leydig cells were the co-occurring keywords with the highest frequency, which reflected the focus of research. The keywords could be classified into 4 categories: three major clusters (age stratification, testicular cells related to mechanisms of spermatogenesis, and biomarkers such as hormones) and one minor thematic cluster (clinical manifestations), which reflected multiple focuses of relevant research. According to **Figures 5B**, **C**, which highlight the networks of “Leydig cells” and “Klinefelter syndrome”, respectively, a robust association was found between Leydig cell function and numerous biomarkers, such as luteinizing hormone, testosterone and INSL3, which are mainly involved in the progression of germ cell failure and molecular dynamics regulation related to KS.

**Figure 5D** displays the same network visualization of the keyword map colored based on the average publication year of keywords to visualize focus transfer in research on Leydig cells in KS patients over time. **Figure 5E** applies density visualization to present the research depth of keywords, **Figures 5F, G** indicate that the areas of intensively researched domains in the field are primarily located in the high-frequency keywords mentioned above.

#### 3.1.4 Reproductive hormone characteristics in literature data

**Figure 6:**
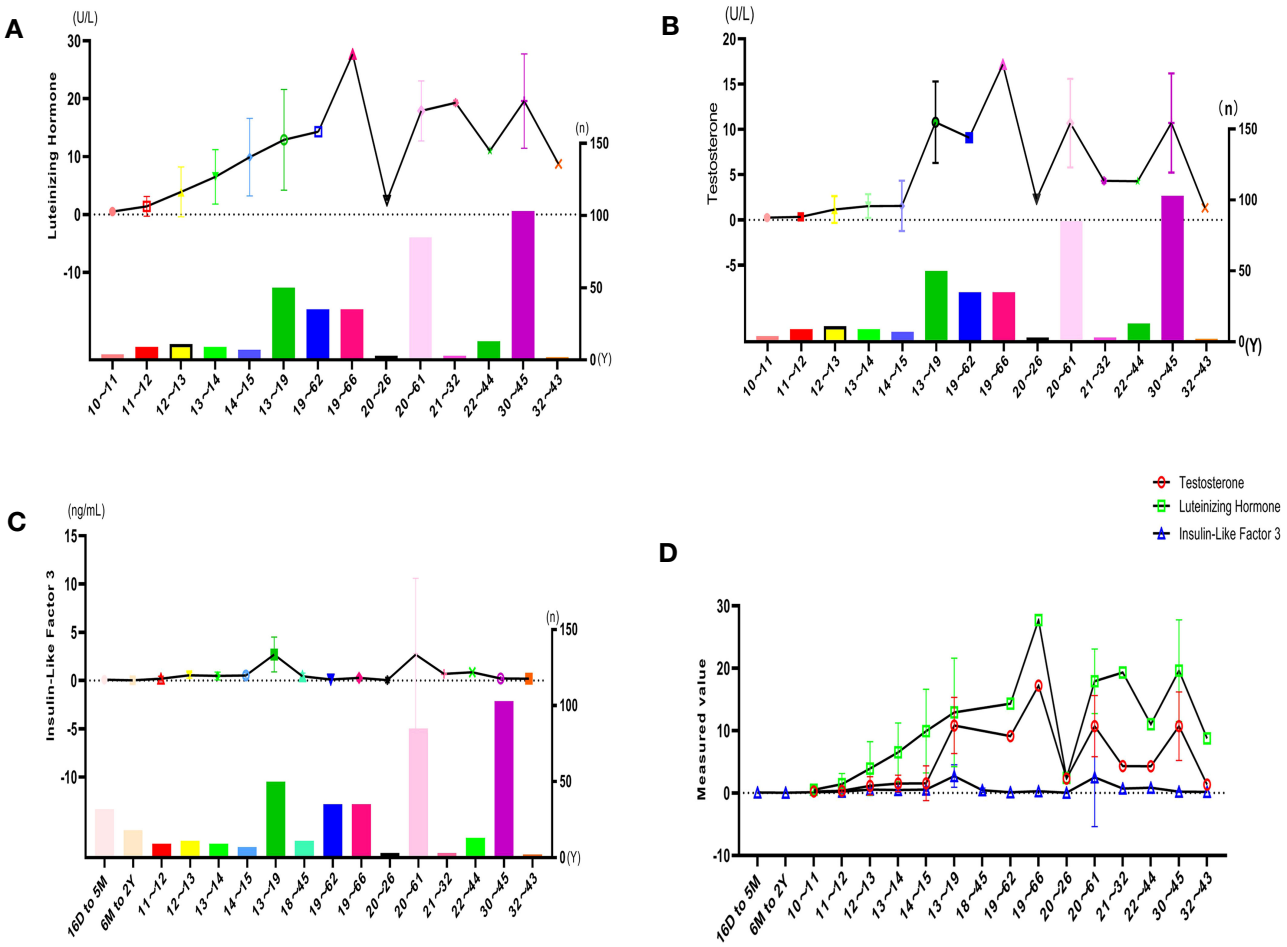
Hormone indicators for quantitative analysis: **(A)** LH characteristics of literature. **(B)** T characteristics of the literature. **(C)** INSL3 characteristics of the literature. **(D)** Reproductive hormone characteristics of literature.

The results of the subgroup analyses of literature data showed that the levels of three hormones in KS patients fluctuated with age, in which the changes in serum testosterone and luteinizing hormone were roughly similar, with peaks in the ages of 13-19 and 30-45, respectively. However, INSL3 was relatively stable, with a peak only at the age of 13-19 (**Figures 6A–D**, **Table 3**). However, the literature data do not provide much information on whether to obtain sperm, so it is impossible to analyze the sperm acquisition rate at different ages, which will be effectively supplemented in our real-world research.

**Table 3:** The Reproductive hormone characteristics of patients in literature data.

Age(D/M/Y)	Sample size	T(nmol/L)				LH(U/L)				INSL3(ng/mL)			
		Mean	Standard deviation	Maximum	Minimum	Mean	Standard deviation	Maximum	Minimum	Mean	Standard deviation	Maximum	Minimum
16 D~5 M	32	NA	NA	NA	NA	NA	NA	NA	NA	0.092	NA	0.222	0.026
6 D~2 Y	18	NA	NA	NA	NA	NA	NA	NA	NA	0.039	NA	0.011	0.11
10~11 Y	4	0.253	0.173	NA	NA	0.5	0.3	NA	NA	NA	NA	NA	NA
11~12 Y	9	0.346	0.223	NA	NA	1.4	1.7	NA	NA	0.2	0.24	NA	NA
12~13 Y	11	1.152	1.481	NA	NA	3.9	4.3	NA	NA	0.55	0.3	NA	NA
13~14 Y	9	1.525	1.315	NA	NA	6.5	4.7	NA	NA	0.48	0.4	NA	NA
14~15 Y	7	1.562	2.775	NA	NA	9.9	6.7	NA	NA	0.54	0.27	NA	NA
13~19 Y	50	10.8	4.5	NA	NA	12.9	8.7	NA	NA	2.7	1.8	NA	NA
20~61 Y	85	10.7	4.9	NA	NA	17.9	5.2	NA	NA	2.5	7.9	NA	NA
22~44 Y	13	4.29	NA	10.81	1.27	2.4	NA	15.6	0	0.06	NA	0.62	0
20~26 Y	3	2.31	NA	3.37	1.27	19.3	NA	19.4	15.5	0.7	NA	0.78	0.12
21~32 Y	3	4.32	NA	4.99	3.69	11	NA	12	9.2	0.86	NA	0.95	0.83
31.5~42.9 Y	2	1.34	NA	2.68	0	8.75	NA	12.8	4.7	0.19	NA	0.31	0.07
19.31~62.35 Y	35	9.1	NA	22.2	0.6	14.3	NA	49.3	0.2	0.12	NA	0.6	0.049
18.97~66.23 Y	35	17.2	NA	27.4	1	27.7	NA	49.2	1.9	0.29	NA	1	0.049
>18 Y	4	NA	NA	NA	NA	NA	NA	NA	NA	0.39	NA	NA	NA
9.9~73.7 Y	82	NA	NA	NA	NA	NA	NA	NA	NA	NA	NA	NA	NA
22.35~41.25 Y	103	10.71	5.48	NA	NA	19.6	8.14	NA	NA	0.225	0.0764	NA	NA
18~45 Y	11	NA	NA	NA	NA	NA	NA	NA	NA	0.440	0.35	NA	NA

D, Days; M, Months; Y, Years; T, Testosterone; LH, luteinizing hormone; INSL3, insulin-like factor 3; NA, not available.

### 3.2 Real-world results

#### 3.2.1 Basic characteristics of patients

**Figure 7:**
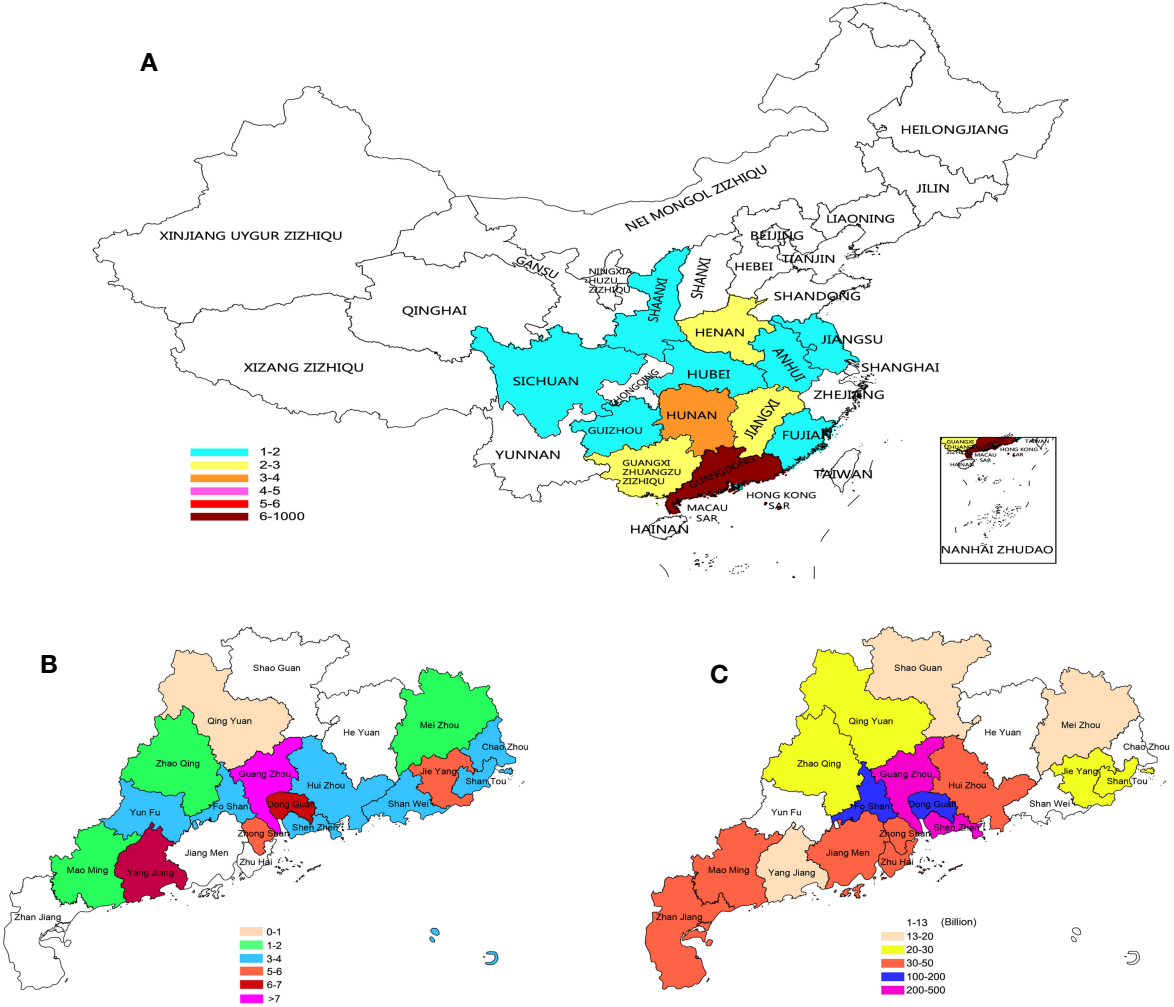
Distribution of KS patients: **(A)** Distribution of KS patients in China. **(B)** KS patients in Guangdong. **(C)** GDP of Guangdong cities in 2021.

The 75 patients with KS were all from different cities in China. Since our hospital is under the jurisdiction of Guangdong Province, the majority of patients in Guangdong Province are less distributed in other regions. Although it cannot reflect the distribution of all of China, it can also reflect certain unbalanced characteristics (**Figure 7A**). In Guangdong Province, there are also significant differences in the distribution of KS; in addition, Guangzhou, Dongguan, Yangjiang, Zhongshan and Jieyang are also highly distributed (**Figure 7B**). We collected the gross domestic product (GDP) of various cities in Guangdong Province in 2021 (Data from http://stats.gd.gov.cn/fsjdgnsczz/content/post_3813633.html), and we found an interesting phenomenon: the distribution of patients with KS seems to have a certain relationship with the distribution of GDP in various cities. The Pearl River Delta region has a relatively high GDP, and the distribution of KS is also relatively dense (**Figure 7C**). There may be some potential correlation between the production relationship and the occurrence of KS, which is worth further exploration. The age of these patients ranged from 19 to 47 years old, with an average of 29.88 ± 4.25 years old; 39 people had used human chorionic gonadotropin and human menopausal gonadotropin hormone before operation, 25 people had used traditional Chinese patent medicines and simple preparations, and 11 people had not used any medicine; 27 sperm were obtained through testicular microscopic sperm extraction, and 48 sperm were not obtained, with a total sperm capture rate of 36.0%.

#### 3.2.2 Tissue transformation and sperm

**Figure 8:**
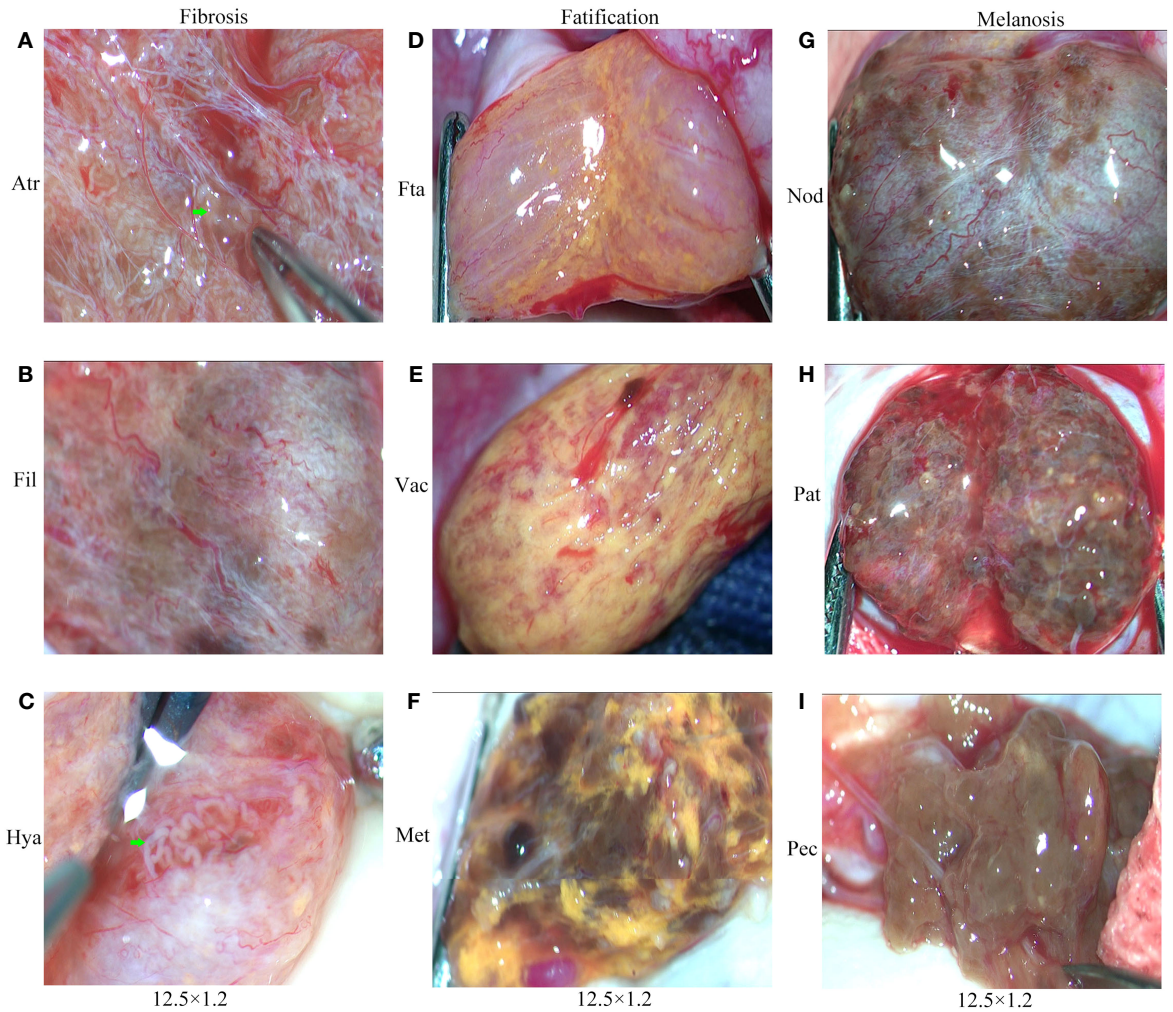
Testicular classification of KS patients. **(A)** Atrophy (Atr). **(B)** Filamentous (Fil). **(C)** Hyaline (Hya). **(D)** Fat accumulation (Fta). **(E)** Vacuolation (Vac). **(F)** Melanoid transformation (Met). **(G)** Nodular (Nod). **(H)** Patchy (Pat). **(I)** Pectinicity (Pec).Green arrow: shaped and spermatogenic tubules.

The intratesticular conditions of 75 patients with mTESE were photographed and recorded by video. We recorded 75 video clips and nearly 1000 photos. Based on the characteristic metabolic changes in testicular tissue of all patients, we divided the testicular tissue of KS patients into three categories for the first time: fibrosis (Fib), fatification (Fat) and melanosis (Mel). Each category can also be divided into three levels according to the degree of change. Fibrosis includes atrophic (Atr), filamentous (Fil), and hyaline (Hya); fatification includes fat accumulation (Fta), vacuolation (Vac), and melanoid transformation (Met); and melanosis includes nodular (Nod), patchy (Pat), and pectinicity (Pec) (**Figures 8A–I**).

**Figure 9:**
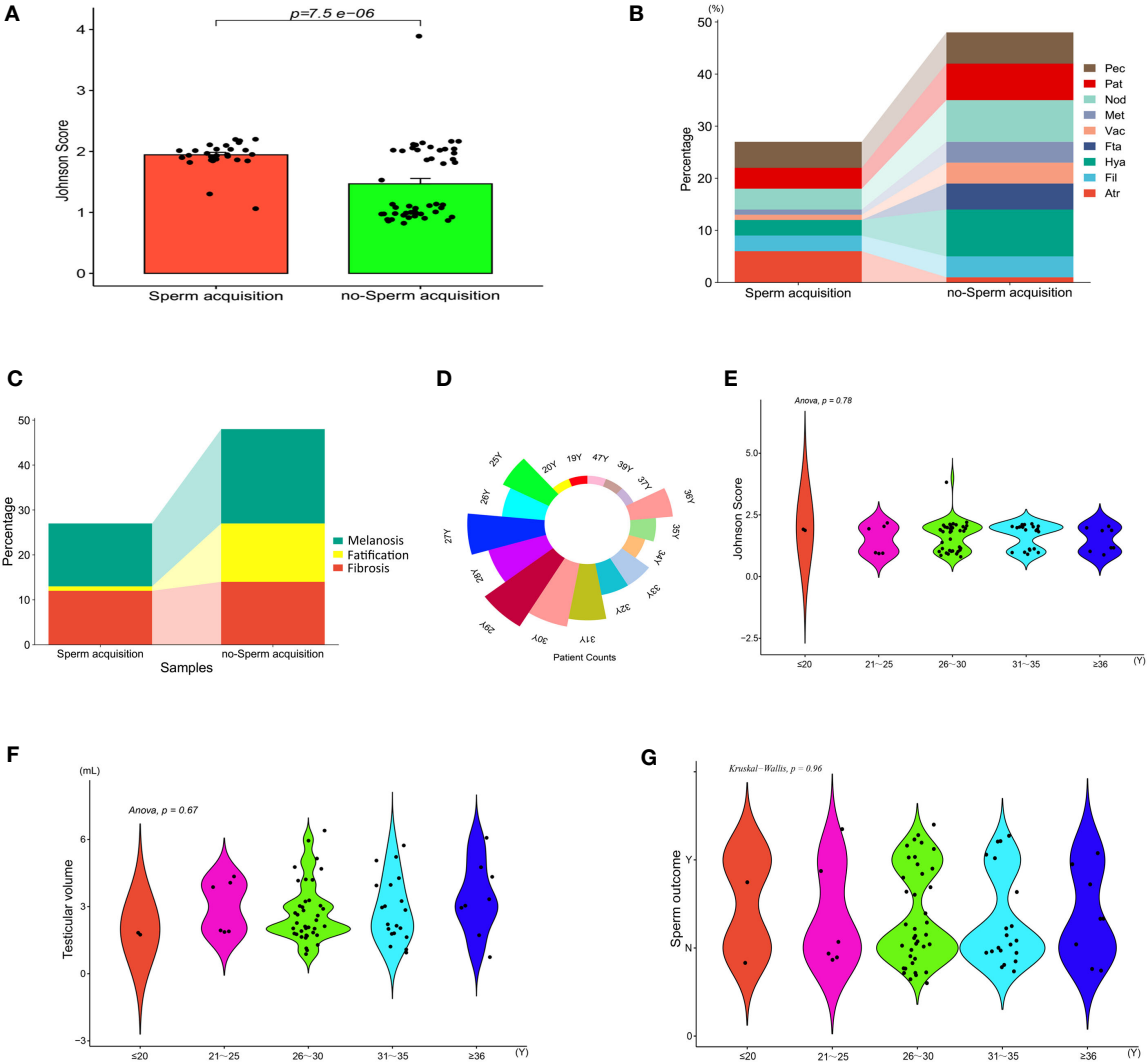
Indicators between KS patients. **(A)** Johnson score. **(B)** Testicular tissue classification. **(C)** General classification of testicular tissue. **(D)** Patient count. **(E)** Johnson score of different ages. **(F)** Testicular volume at different ages. **(G)** Sperm outcome of different ages.

Referring to the classification criteria and Johnson score, we also compared the testicular tissue status and pathological changes between those who obtained sperm during mTESE and those who did not. The results showed that the Johnson score of those who obtained sperm was significantly higher than that of those who did not obtain sperm (**Figure 9A**, **Table 4**). Of the 27 sperm recipients, 12 had fibrosis, 1 had fatification, and 14 had melanosis. Among the 48 patients without sperm acquisition, there were 14 cases of fibrosis, 13 cases of fatification, and 21 cases of melanosis. There was a significant difference between the two groups (**Figure 9B**) (*χ^2 = ^* 6.467, *p*=0.039). The sperm acquisition rate of fibrosis was 46.15%, that of fatification was 7.14%, and that of melanosis was 40.00%. It was difficult for fatification to obtain sperm (**Figure 9C**).

**Table 4:** The characteristics of KS patients with and without sperm.

Group	Sample size (n)	Age (Y)	FSH(mIU/mL)	LH(mIU/mL)	T (nmol/L)	E_2_ (pmol/L)	Left testicular volume (mL)	Right testicular volume (mL)	Johnson Score
With sperm	27	29.37 ± 3.94	31.43 ± 14.09	17.66 ± 7.19	7.50 ± 4.55	68.44 ± 39.10	3.26 ± 1.40	3.26 ± 1.40	1.94 ± 0.21
Without sperm	48	30.17 ± 4.42	34.95 ± 16.83	21.97 ± 8.92	5.33 ± 34.00	59.93 ± 3.48	2.60 ± 1.23	2.58 ± 1.25	1.47 ± 0.61
p	—	0.439	0.36	0.035	0.023	0.328	0.039	0.035	<0.001

FSH, follicle-stimulating hormone; T, Testosterone; LH, luteinizing hormone; E_2_, estradiol.

Based on age as a hierarchical basis and every five years as a stage, we compared the tissue changes and Johnson scores of patients of different age groups, with age divided into five stages: ≤ 20, 21-25, 26-30, 31-35, and ≥ 35 (**Figure 9D**). Univariate AVNOA analysis revealed that there was no difference in testicular volume, mean Johnson score, or sperm outcome among patients of different ages (**Figures 9E–G**).

#### 3.2.3 Hormones metabolism and sperm

**Figure 10:**
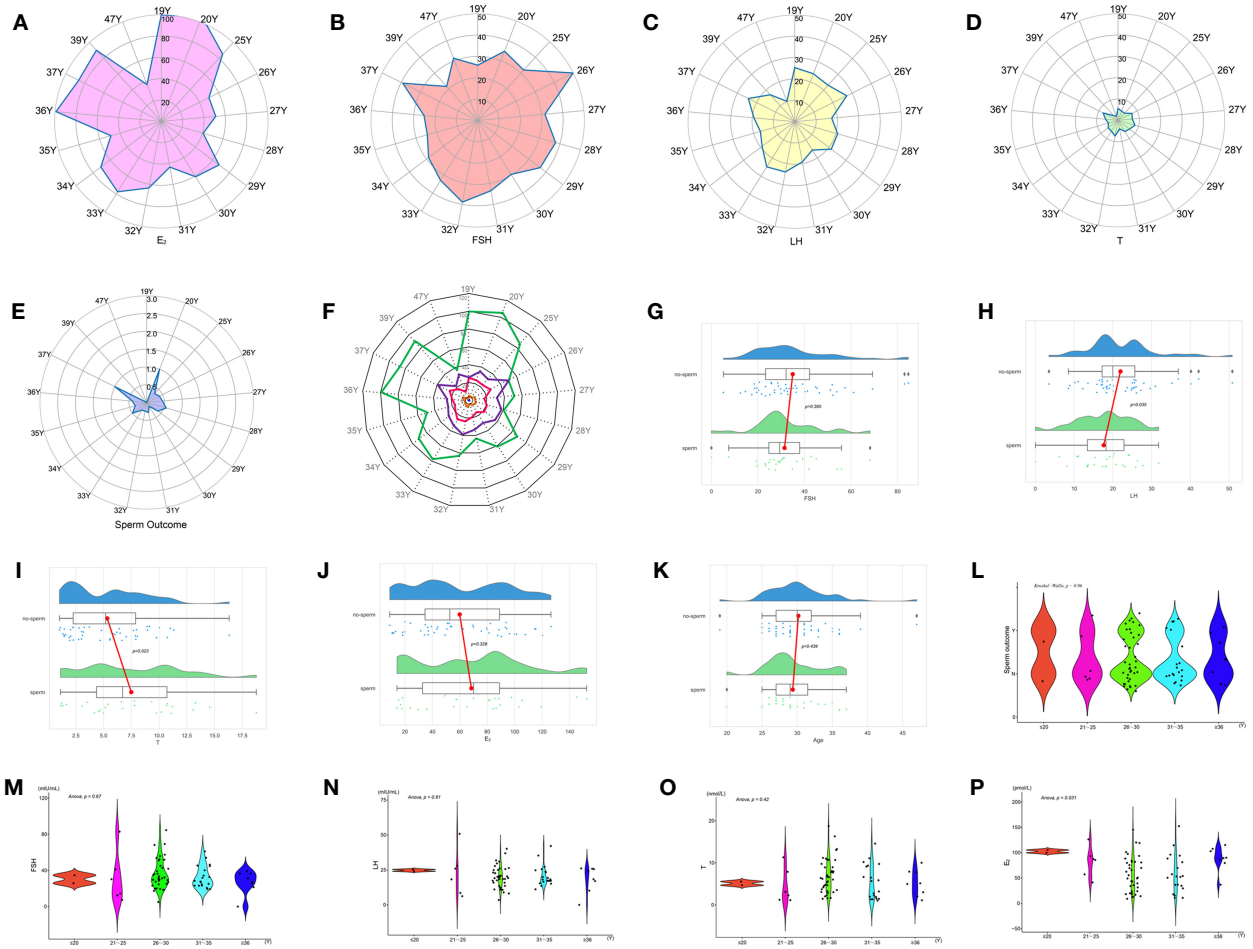
Indicators between KS patients of different ages: **(A)** E_2_. **(B)** FSH. **(C)** LH. **(D)** T. **(E)** Sperm outcome. **(F)** Comprehensive indicators. **(G)** FSH. **(H)** LH. **(I)** T. **(J)** E_2_. **(K)** Age. **(L)** Sperm outcome. **(M)** FSH. **(N)** LH. **(O)** T. **(P)** E_2_.

According to the age sequence, taking the mean value of patients of the same age for analysis of reproductive hormone indicators in patients with KS at different ages, it was found that FSH had a high peak at 26, 32, and 37 years of age, LH had a peak at 19, 26, 33, and 37 years of age, T had a peak at 19, 28, 32, and 37 years of age, and E_2_ had a high peak at 19, 20, 36, and 37 years of age. At the ages of 20, 28, 34, and 37, the probability of mTESE acquiring sperm had a peak value (**Figures 10A–F**).

Based on the principle of obtaining sperm or not, comparing the reproductive hormones of those who obtained sperm with those who did not, the results showed that the LH of those who obtained sperm was significantly lower than that of those who did not obtain sperm, and T was significantly higher than that of those who did not obtain sperm, while there was no significant difference in age, FSH, and E_2_ (**Figures 10G–K**, **Table 4**).

Age was used as the basis for stratification, and every 5 years was used as a stage. Age is divided into five stages: ≤ 20, 21-25, 26-30, 31-35, and ≥ 35. Single-factor ANOVA revealed that there was no difference in sperm outcome, FSH, LH, and T among patients of different ages, but there was a significant difference in E_2_ (**Figures 10L–P**).

### 3.2.4 Cells metabolism and sperm

**Figure 11:**
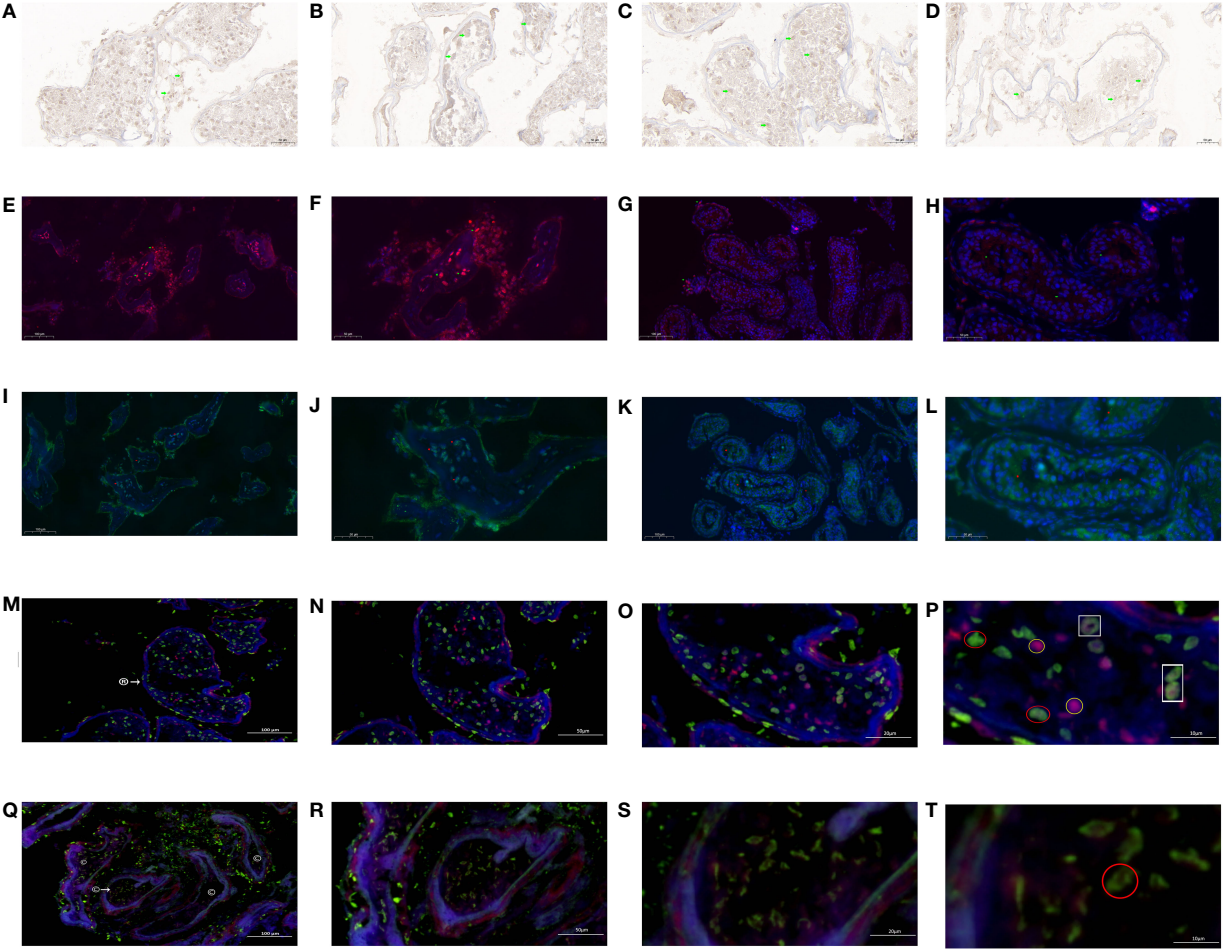
INSL3 receptor and androgen receptor in KS patients. **(A)** INSL3 between seminiferous tubules (green mark). **(B)** INSL3 in seminiferous tubules (green mark). **(C)** INSL3 in the seminiferous tubule of spermatozoa (green mark). **(D)** INSL3 in the seminiferous tubule without sperm (green mark). **(E)** INSL3 in the seminiferous tubule of spermatozoa (green mark, red fluorescence, 100×). **(F)** INSL3 in the seminiferous tubule of spermatozoa (green mark, red fluorescence, 200×). **(G)** INSL3 in the seminiferous tubule without sperm (green mark, red fluorescence, 100×). **(H)** INSL3 in the seminiferous tubule without sperm (green mark, red fluorescence, 200×). **(I)** Androgen receptor in the seminiferous tubule of spermatozoa (red mark, green fluorescence, 100×). **(J)** Androgen receptor in the seminiferous tubule of spermatozoa (red mark, green fluorescence, 200×). **(K)** Androgen receptor in the seminiferous tubule without sperm (red mark, green fluorescence, 100×). **(L)** Androgen receptor in the seminiferous tubule without sperm (red mark, green fluorescence, 200×). **(M)** The androgen receptor and INSL3 receptor in the seminiferous tubule of spermatozoa: ^®^: find the sperm seminiferous tubule. Green fluorescence: INSL3 receptor. Red fluorescence: androgen receptor. 100×. **(N)** The androgen receptor and INSL3 receptor in the seminiferous tubule of spermatozoa: Green fluorescence: INSL3 receptor. Red fluorescence: androgen receptor. 200×. **(O)** The androgen receptor and INSL3 receptor in the seminiferous tubule of spermatozoa: Green fluorescence: INSL3 receptor. Red fluorescence: androgen receptor. 500×. **(P)** The androgen receptor and INSL3 receptor in the seminiferous tubule of spermatozoa. INSL3 receptor: green fluorescence, marked in red circle. Androgen receptor: red fluorescence, marked in yellow circle. Overlapping androgen receptor and INSL3 receptor: marked in white box. 1000×. **(Q)** The androgen receptor and INSL3 receptor in the seminiferous tubule without sperm: ^©^: the seminiferous tubule without sperm. Green fluorescence: INSL3 receptor. Red fluorescence: androgen receptor. 100×. **(R)** The androgen receptor and INSL3 receptor in the seminiferous tubule without sperm: Green fluorescence: INSL3 receptor. Red fluorescence: androgen receptor. 200×. **(S)** The androgen receptor and INSL3 receptor in the seminiferous tubule without sperm: Green fluorescence: INSL3 receptor. Red fluorescence: androgen receptor. 500×. **(T)** The androgen receptor and INSL3 receptor in the seminiferous tubule without sperm: INSL3 receptor: green fluorescence, marked in red circle. 1000×.

Immunohistochemistry showed that the brown-loving INSL3 receptor was mainly deposited in the seminiferous tubule wall (**Figure 11A**) and inside the tubule (**Figure 11B**). The concentration of INSL3 deposition in the seminiferous tubules with sperm (**Figure 11C**) was significantly higher than that in the seminiferous tubules without sperm (**Figure 11D**).

Immunofluorescence (single label) labeling showed that the INSL3 receptor in the wall and lumen of the seminiferous tubule where sperm were found (**Figures 11E, F**) was significantly higher than that in the seminiferous tubule where no sperm were found (**Figures 11G, H**). Similarly, the androgen receptor in the wall and lumen of the sperm seminiferous tubule (**Figures 11I, J**) was significantly higher than that in the seminiferous tubule without sperm (**Figures 11K, L**).

To confirm whether the INSL3 receptor and androgen receptor are expressed in the same cell, we conducted immunofluorescence double labeling tests and observed typical regions at different magnifications of 100×, 200×, 500×, and 1000×. The results showed that the INSL3 receptor and androgen receptor (**Figures 11M–O**) in the seminiferous tubule of spermatozoa were significantly higher than those in the seminiferous tubule without spermatozoa (**Figures 11Q–S**), while the INSL receptor between the seminiferous tubule of spermatozoa was significantly lower than those in the seminiferous tubule without spermatozoa. After gradual magnification, it was found that the INSL3 receptor and androgen receptor in the sperm seminiferous tubule were roughly the same, and individual INSL3 receptors and androgen receptors might overlap on the same cell (**Figure 11P**). However, no serious imbalance was found between the two in the sperm seminiferous tubule. Only the INSL3 receptor was found, while the androgen receptor was missing (**Figure 11T**).

## 4 Discussion

As the most common sexual chromosome aneuploidy abnormality in men, the cause of KS has always been a complex matter. Although there have been many epidemiological studies since the discovery of KS for more than 60 years, no definitive conclusions have been reached. Currently, there are many opinions that the occurrence of KS is the result of gene mutation, but what causes the mutation? However, this is not yet known. An increasing number of studies have confirmed that changes in the Earth’s environment also affect changes in human genetic material, resulting in a large number of chromosomal aneuploidies. As a special type of sex chromosome aneuploidy, the main reason for its occurrence may have a potential relationship with changes in the Earth’s environment. For the first time in our study, we analyzed the distribution of birthplaces of KS patients. Although our number of cases is small, they come from various regions in China and exhibit different distribution patterns. In particular, the distribution of patients in Guangdong Province is also very uneven. We compared the gross domestic product (GDP) of the cities where these patients reside in 2021 (Data from http://stats.gd.gov.cn/fsjdgnsczz/content/post_3813633.html) and found that the distribution of KS patients in Guangzhou and Dongguan with higher GDP was also higher. Although we cannot trace whether the GDP of these regions was at a high level when these patients were born, from the perspective of Jieyang and Yangjiang, where the industrialization process is relatively slow, their KS distribution is also higher. At the time of the birth of these patients, these two cities were at the beginning of the industrialization process. Therefore, we speculate that there may be some inherent links between the industrialization process and the occurrence of KS. In future research, we may pay closer attention to the relationship between the occurrence of KS and industrialization.

By means of the bibliometric analysis of 105 documents included and the mapping of the scientific landscape of KS and Leydig cells, we analyzed the countries, journals and authors contributing to this domain and identified some focus topics worth continuous and in-depth exploration for our follow-up studies. From the perspective of the general trend of publications, research on KS and Leydig cells, with erratic growth in annual output, has gradually emerged among academic fields since the 1990s. In particular, the annual publication output reached its peak in the past decade, which suggested that the topic had come into the spotlight with certain research heat and depth over time. The reason for the explosion might be that thanks to the improvement in the diagnostic rate of KS, researchers have an urge to further determine the related internal pathogenesis and mechanisms of germ cell development through pathological histochemistry or burgeoning technologies such as single-cell transcriptomic analysis.

As the geographic visualization and data analysis showed, international cooperation has been primarily conducted in the countries with the largest output of relevant publications, although the regional distribution is dispersed. In the analysis of clustering, temporal and density distribution of keywords in cooperation network, we determined the distribution and internal relationship of hotspots in the field. The keywords with the highest frequency are “KS”, “Leydig cells”, “testosterone”, “inferiority”, “Sertoli cells” and “boys and men”, which indicates that the function of testicular somatic cells is the center of research in this field and that there is a highly connected interaction network between KS and Leydig cells. Whether disordered Leydig cell maturation or later pathological hyperplasia might be associated with testicular failure, ultimately leading to the general clinical characteristic of infertility in KS patients.

Combined with the temporal network clustered by average publication date, we found that the age of KS patients and serological biomarkers have gradually attracted increasing attention from scholars over time. Numerous studies have valued the variation in testosterone, luteinizing hormone (LH) and insulin-like factor 3 (INSL3, regarded as a promising biomarker of Leydig cell maturation) in KS patients of different age cohorts and aimed to use the changing pattern of these indexes as a reflection of the functional status of somatic cells in seminiferous tissue, as well as the prediction of testicular spermatogenesis degeneration (35, 36). For instance, these biomarkers might assist clinicians in choosing the appropriate opportunity for micro sperm extraction and judging the clinical outcome of hormone therapy (37). These might be the most recent topics that suggest future trends in the domain.

Overall, we used the rigorous method of bibliometric analysis to obtain the above valuable information and systematically perused the literature that has a significant impact on the research status and trends in the field. According to the influence of countries and authors, the cited situation of the articles and the frequency of the keywords in the studies within the past three decades (1992-2022), we found that the unstable annual publication output, scattered regional distribution and unsatisfactory scholar cooperation might lead to a lack of an intact theoretical framework in this field, but it has been gradually acknowledged and emphasized that there is a strong link between KS and Leydig cells, which will be one of the core topics for KS. Morphological proliferation, insufficiency of function or disorder of apoptosis regulation in Leydig cells might be tightly related to testicular function at different ages of KS and ultimately show changes in serum markers such as testosterone, LH and INSL3. Eventually, this bibliometric analysis provides suggestions and approaches for the following subgroup analysis and real world research, by which we expect to determine a reasonable and considered method for fertility optimization and preservation of KS patients.

The production and apoptosis of cells are fundamental to the cell cycle. As the basis of human reproduction, sperm also follow the principles of all things. Research shows that the natural production and apoptosis cycle of human sperm is approximately 3 months (38, 39). Bhanmeecho et al. conducted a group study of 55 dogs of different ages and found that the degree of testicular interstitial fibrosis in elderly dogs was significantly higher than that in other age groups. With increasing age, there was a significant difference in the degree of spermatogenic cell degeneration. Age was positively correlated with interstitial fibrosis and positively correlated with the degree of testicular tubular atrophy (40). Therefore, age is considered to be one of the most critical factors regulating spermatogenesis.

Research has found that aging of the gonads of men over the age of 40 can significantly reduce their fertility, including not only changes in sperm quality and sperm function but also a reduction in the ability to combine sperm and testis (41). As the center of the male reproductive system, testicular dysfunction, oxidative stress damage, apoptosis, inflammation, and decreased immune function all contribute to the cessation of spermatogenesis (42). However, there were also opposing views. Mularoni et al. found that with age, the number of Leydig cells in the testis decreased, but the peripheral serum androgen concentration did not significantly decrease (43). Research has shown that there are two different types of Leydig cells in mammalian testes: fetal Leydig cells (FLCs) and adult Leydig cells (ALCs), which appear before and after birth, respectively. Among them, FLCs contain a large number of lipid droplets, while ALCs contain a small amount of lipid droplets. When FLCs fail to degrade normally after birth and still exist in the adult testicles, it can affect the function of ALCs in late adolescence (44). Therefore, to clarify the relationship between statue of Leydig cells disorder and spermatogenesis in patients with KS at different ages might be helpful in changing the fertility of patients with KS, We suspect that the proportion of FLCs may be significantly higher than that of ALCs in KS patients with transitional steatosis of the testicular interstitium, resulting in decreased local spermatogenic function, which may also be the main reason for their low mTESE sperm acquisition rate.

Unlike previous scholars, we found significant differences in reproductive hormone indicators among patients with KS at different ages, with FSH having a peak at 26, 32, and 37 years; LH having a peak at 19, 26, 33, and 37 years; T having a peak at 19, 28, 32, and 37 years; and E_2_ having a peak at 19, 20, 36, and 39 years. There were also differences in whether there was focal spermatogenesis in the testicles of KS patients at different ages, with the probability of recovering sperm through mTESE reaching a peak at the ages of 20, 28, 34, and 37. The LH of patients with sperm acquisition was significantly lower than that of patients without sperm acquisition, and the T was significantly higher than that of patients without sperm acquisition. There were also significant differences in E_2_ among patients of different ages.

This phenomenon is greatly different from the view of other scholars that the younger a KS patient is, the easier it is to obtain sperm (45, 46). Our analysis may have the following reasons: (1) Our small sample size for each age group may cause bias in the results. (2) Our research population was mainly older adults, not adolescents, so the population composition may have some interference with the results. (3) Although we may have the first two types of interference, from the perspective of the probability of mTESE sperm recovery in men of childbearing age, at least the viewpoint of scholars in the early and middle stages of childbearing age is not applicable, which also suggests that the spermatogenesis of men of childbearing age does not develop according to the rule of age, and there may be other regulatory factors in the spermatogenesis of patients of KS childbearing age, and such factors can be interfered with. This may also be a breakthrough in solving the bottleneck in the treatment of KS spermatogenesis and provide some inspiration for our further research.

In the past, the histopathological Johnson score has been considered the gold standard for evaluating testicular spermatogenesis (48). However, with the development of microscopic testicular sperm extraction (mTESE), an increasing number of clinical practices have confirmed the presence of focal spermatogenesis in the testicles of patients with nonobstructive azoospermia (49, 50). In previous studies related to mTESE, we have also found that the sperm recovery rate of patients with nonobstructive azoospermia undergoing mTESE surgery can reach 38.7% (51). KS is a typical nonobstructive azoospermia with congenital chromosomal aneuploidy defects, but spermatogenesis may still exist in its testicles (52, 53). Similar to studies by other scholars, we also found focal spermatogenesis in infertile patients with KS, with a mTESE sperm recovery rate of 36.0%. Although this number is lower than that reported by other scholars, this is accurate data, which may be related to the patient’s sample size, age, and previous treatment history. The sample size of this study is small, the age is concentrated in the reproductive age period, and all patients have not received any treatment three months before surgery, or a 3-month washout period is ensured after receiving treatment. Therefore, the mTESE sperm recovery rate in this study will be low.

On the other hand, the Johnson score of testicular tissue in all patients with KS in this study remained between 1-2 points, while only one of 75 patients had a score of 4. According to the Johnson scoring standard, the lower the score, the poorer the development of testicular seminiferous tubules and the lower the rate of spermatogenesis. Therefore, this may also be one of the main factors that caused the sperm acquisition rate in this study to be lower than that of other scholars. Our comparison of Johnson scores between patients with and without sperm also confirms this phenomenon, and the Johnson scores of patients with sperm are significantly higher than those of patients without sperm.

However, from a single case of histopathological Johnson score, we cannot obtain a valuable predictive conclusion on the presence or absence of focal spermatogenesis from a patient’s Johnson score. Therefore, we believe that conducting testicular biopsy in patients with KS before mTESE is meaningless, as it can lead to damage to the testicular microenvironment and is not conducive to the development of focal spermatogenesis.

The hypothalamus-pituitary-testicular gonadal axis is considered to be the center for regulating spermatogenesis (54, 55). Our previous studies have also found that the concentration of reproductive hormones in the testicular environment also plays a key role in regulating spermatogenesis (51). Therefore, reproductive hormones should have a crucial role in spermatogenesis in patients with KS. Due to the presence of X-chromosome aneuploidy, patients with KS are clinically characterized by high levels of follicle stimulating hormone and luteinizing hormone, accompanied by low or normal testosterone. Their follicle-stimulating hormone and luteinizing hormone levels can exceed the normal range by 2-3 times or even higher (56). It has been reported that lowering FSH and LH can promote spermatogenesis in patients with nonobstructive azoospermia (57), but the effect on KS patients is unclear. Therefore, exploring the rules of reproductive hormones in patients with KS has a guiding role in regulating spermatogenesis.

Previous studies have found that Leydig cells appear and can secrete androgens at the 8th week of a male embryo. At the 9th to 14th week of the embryo, Leydig cells turn into differentiated cells. At the 15th to 18th week of the embryo, Leydig cells mature and have a strong androgen secretion ability, forming a peak of androgen secretion during the embryonic period. Thereafter, they gradually degenerate until puberty before increasing and increasing the secretion of androgens again (58–60). Currently, it is believed that there are a large number of LH receptors on the surface of Leydig cells, which, after binding with LH, initiate the transformation process of neutral fatty acids and cholesterol lipids in Leydig cells, promote the conversion of cholesterol into testosterone, keep the epithelium of spermatogenic tubules close to the lumen in a long-term high concentration of testosterone, promote the differentiation of late spermatocytes, and complete the spermatogenesis process (61, 62). There may be a local “short loop” regulatory pattern between the functions of mesenchymal cells and Sertoli cells, and estradiol secreted by Sertoli cells may interfere with the expression of receptors on the surface of mesenchymal cells (63, 64). Therefore, analyzing the function of Leydig cells is crucial for promoting spermatogenesis.

In the subgroup analysis following the bibliometric analysis, we extracted the indicators testosterone, LH, and INSL3 related to Leydig cells and found that the levels of these hormone or hormone-like indicators in patients with KS fluctuated with age, especially when the levels of testosterone, LH, and INSL3 in men with KS were observed to be different at various age groups, and the peaks also occurred at different ages. The change trend of serum testosterone and LH is generally similar, reaching a peak at the ages of 13-19 and 30-45, respectively. Comparatively, INSL3 is relatively stable, peaking only at the age of 13-19, and the peaks of testosterone and INSL3 significantly lag behind those of LH. Correspondingly, in our cross-sectional study of men of childbearing age, we found that the peak ages of testosterone and LH were also similarly reached at the ages of 19, 26-28, 32-33, and 37. The age cohort with higher success rates in sperm extraction was also concentrated at 20 and 37 years old. In the same age group, lower LH and higher testosterone may be associated with positive spermatogenic function. This indicates that the fluctuations of hormones in patients with KS at childbearing age follow a certain rule, which might be related to the development status of testicular spermatogenic tissue, the maturity of somatic cells adjacent to germ cells, and sensitivity to the hormone-stimulating effect at different ages.

Previous studies (9, 32, 35) have shown that during childhood and even early puberty of KS, pituitary-gonadal function is normal, and testicular biopsies of prepubertal KS boys show that the number of germ cells is reduced, but the seminiferous tubules are preserved and Sertoli cells and Leydig cells still work regularly, which indicates that hormone synthesis and secretion may be normal. From the middle of puberty, the seminiferous tubules of patients have rapidly accelerated degradation, with varying degrees of fibrosis, transparency, and interstitial proliferation, as well as maturation disorders and functional deficiencies of Sertoli cells and Leydig cells. However, in this study, LH, FSH, and other hormones in patients with KS still fluctuated during childbearing age and did not directly increase to high levels of gonadotropins, while testosterone levels dropped to low or subnormal levels after an initial increase and were still able to reach a new peak. The reason for these phenomena may be related to the residual spermatogenic tissue in the testis, and it might also be because the stimulation response of LH-testosterone may still exist at a specific childbearing age and stimulates the spermatogenesis of the remaining spermatogenic tissue, which shows that KS at childbearing age still has the potential to produce sperm. In other words, not all somatic cells decline irreversibly with age, and the remaining cells may have the potential to perform compensatory functions, but this needs to be verified in further research. Thus, we believe that the evolution of the morphology and function of somatic cells such as Leydig cells in the testicles of KS of childbearing age and the generated hormone fluctuations are of great significance for research related to the pathogenesis and progression of KS, which also provides a novel explanation for the controversial topic of the relationship between testicular spermatogenic function and hormones.

In our study, we found that LH in sperm recipients was significantly lower than that in nonsperm recipients, and T was significantly higher than that in nonsperm recipients. There were also significant differences in E_2_ among patients of different ages. The differences among the three have once again confirmed the relationship between spermatogenesis and the LH——T——E_2_ regulatory axis. We believe that LH activation of testicular Leydig cells to secrete sufficient T is the basis for stimulating spermatogenesis, but the balance of transformation between T and E_2_ is also a necessary condition for ensuring spermatogenesis, and none of the three is indispensable. We have also confirmed through immunofluorescence single and double labeling that the proportion of androgen receptors and INSL3 receptors is relatively coordinated in the testicular tissue around the seminiferous tubules with normal spermatogenesis, while in the testicular tissue around the seminiferous tubules without spermatogenesis, there is a serious imbalance in the proportion of androgen receptors and INSL3 receptors. This phenomenon is the first time that it has been discovered. Unlike previous studies, we found that androgen receptors and INSL3 receptors are more intensively distributed in the wall and inside the seminiferous tubules. These two receptors are jointly expressed on the surface of supporting cells. In local areas of transitional proliferation of interstitial tissue, there is a high concentration of androgen receptors, while INSL3 receptors are not widely distributed. These areas are also the areas where interstitial tissue degeneration is most severe, and there is even the possibility of mesenchymal neoplasia. Therefore, we believe that the ratio of INSL3 combined with T may be more suitable as an accurate indicator for predicting spermatogenesis in patients with KS.

## 5 Conclusion

There are significant differences in LH, T, and E_2_ levels between KS patients with focal spermatogenesis at different ages and those without spermatogenesis. The age points of KS patients with easy access to sperm are 20, 28, 34, and 37 years old. The INSL3 receptor and androgen receptor are centrally expressed in the walls and inside the spermatogenic tubules, and a balanced of these metabolites is an important basis for ensuring spermatogenesis. Abnormal metabolism of Leydig cells led to imbalanced expression of INSL3 and androgen receptors, which might be a potential target for spermatogenesis in KS.

### 5.1 Shortcomings

We found a certain correlation between abnormal lipid metabolism in interstitial cells of KS patients and focal spermatogenesis, but there are still the following shortcomings: (1) Our sample size is small and cannot fully verify the condition of KS patients at each age group. It is also necessary to obtain more detailed information of population data in the future in order to make a more accurate map of the population distribution characteristics of KS patients. (2) Although we have made every effort to provided the fullest presentation of the available data and statistical details to support our conclusion, our results may be disrupted by unavoidable potential biases and statistical limitation, which is supposed to be improved in the future large-scale study. (3) There is lack of validation of animal models and cannot simulate the occurrence of abnormal lipid metabolism in KS patients. (4) We need to further explore the mechanism of abnormal lipid metabolism in KS patients.

## Data availability statement

The original contributions presented in the study are included in the article/supplementary material. Further inquiries can be directed to the corresponding author.

## Ethics statement

The studies involving humans were approved by Ethics Committee of Guangdong Provincial Reproductive Science Institute (Guangdong Provincial Fertility Hospital) (Approval No.2021(08)). The studies were conducted in accordance with the local legislation and institutional requirements. Written informed consent for participation in this study was provided by the participants’ legal guardians/next of kin.

## Author contributions

HuL: Funding acquisition, Methodology, Project administration, Writing – original draft, Writing – review & editing. ZHZ: Data curation, Formal Analysis, Investigation, Methodology, Software, Writing – original draft. YG: Conceptualization, Investigation, Resources, Validation, Writing – original draft. HaL: Data curation, Formal Analysis, Investigation, Methodology, Resources, Writing – original draft. ZYZ: Conceptualization, Investigation, Methodology, Writing – original draft. HZ: Conceptualization, Investigation, Methodology, Writing – original draft. WY: Writing – original draft. ZL: Conceptualization, Investigation, Methodology, Writing – original draft. ZQ: Conceptualization, Investigation, Methodology, Writing – original draft. QX: Conceptualization, Investigation, Methodology, Writing – original draft. LH: Investigation, Writing – review & editing. YZ: Methodology, Writing – review & editing. XZ: Methodology, Writing – review & editing.
